# Editorial: Aspiring for onco-functional utopia via radiosurgery

**DOI:** 10.3389/fsurg.2023.1207572

**Published:** 2023-05-16

**Authors:** Harsh Deora, Manjul Tripathi, Jason P. Sheehan

**Affiliations:** ^1^ Department of Neurosurgery, National Institute of Mental Health and Neurosciences, Bengaluru, India; ^2^ Department of Neurosurgery, Postgraduate Institute of Medical Education and Research, Chandigarh, India; ^3^ Department of Neurological Surgery, University of Virginia Health System, Charlottesville, VA, United States

**Keywords:** gamma knife radiosurgery, neurooncology, functional outcome, functional neurosurgery, stereotactic radiosurgery

**Editorial on the Research Topic** Aspiring for onco-functional utopia via radiosurgery

In the history of neurosurgery, neurosurgeons of the 60’s to 90’s may be considered the real heroes. For the first time, neurosurgery noticed that someone could make meaningful changes in the natural history of certain disorders. This became possible because of slow but gradual improvement in technology both in radiology and neurosurgical armamentarium. However, with this, there was a significant increase in the morbidity and mortality profile because anything alien to the brain was considered worthy of resecting out, even at the cost of complications. Frustrated with existing results, Professor Lars Leksell envisioned the stereotactic radiosurgery (SRS) concept to achieve the balance between cure and control, survival, and quality of life (1). SRS has shifted the paradigm in managing most intracranial pathologies, primarily vascular malformations, skull base disorders, and metastases (2). With more than five-decade experience and sufficient evidence, SRS is the treasure of knowledge that is only refined and redefined with each passing day.

This dedicated issue on radiosurgery aimed at obtaining onco-functional balance has five unique articles. Burke et al. illustrated that patients with an aggregate tumor volume greater than 6.5 cc from metastatic melanoma were at increased risk of intracranial death and radiation necrosis when treated with SRS and immune checkpoint therapy. Melanoma, the fifth most common cancer in the USA, with a high propensity for intracranial metastasis, is a significant disease burden. In their large retrospective series, the authors have analyzed that SRS with immune checkpoint inhibitor therapy has become the standard of care for brain metastasis, with an acceptable complication profile and no difference in overall survival, intracranial death, or radiation necrosis concerning the type of immunotherapy, timing of SRS in immunotherapy, number of SRS courses, SRS doses, or number of cumulative lesions treated. Lv et al., in their interesting case report, implanted the Ommaya reservoir in solitary cystic brain metastasis followed by SRS. Though they needed repeated cystic fluid aspiration, the intracranial tumor was effectively and satisfactorily controlled during the follow-up period of 14 months. This strategy may be considered for cystic lesions situated in deep-seated eloquent locations where surgical morbidity and mortality profile might be significant; however, the need for repeated aspirations and chances of blockage of Ommaya are deterrents for making it a common practice.

Aldakhil and Mathieu have illustrated a rare abscopal effect leading to the complete disappearance of extensive meningiomatosis after GKRS. Commonly considered an immunological reaction following conventional radiation, this is a unique report showing the complete disappearance of meningiomas when only the petroclival part was treated earlier. We believe this finding needs to be reported frequently to analyze this interesting yet poorly understood phenomenon's exact prevalence. In another interesting article, Piper et al. tried to answer the difficult question of the management of dural tail. It's been a long, contentious issue if the dural tail is a mere inflammatory response to meningioma or an extension of the tumor. So far, the literature is fractured on defining the dural tail and its management. However, the nodular dural tail is considered an extension of the tumor, and many authors have previously emphasized the need for its radiation even at the cost of likely complications. This research mentions that radiation of the dural tail does not reduce recurrence rates. An improved understanding of dural tail pathophysiology, tumor grade, tumor spread, and radiation response is needed to predict meningiomas' response to radiotherapy better.

Lehrer et al., in their thought-provoking article on preoperative SRS in the management of brain metastasis and gliomas, have extensively evaluated the concept of pre- versus post-surgery SRS in the context of risk of leptomeningeal disease, local control, feasibility, and complication profile. Though presurgery SRS has been practiced for metastasis for quite a long time, its recent evaluation for high-grade gliomas and glioblastoma marks is an interesting take. Preoperative SRS has specific immunogenic effects, which are under evaluation in the ongoing trials.

All the mentioned reports highlight the ongoing pattern of changing radiation practices in complex neurosurgical disorders. These endeavors aim to achieve an onco- functional balance with minimum discomfort to the patient while gaining the best local and systemic control. A better understanding of SRS has empowered neurosurgeons to embark on uncharted seas and fractionation schemes. This issue provides insight into indications, likely management options, and future challenges.
